# Supplementary figures and images for: Short-term microplastic effects on marine meiofauna abundance, diversity and community composition
Source: PeerJ. 2024 Jul 31;12:e17641. doi: 10.7717/peerj.17641 (PMC11297435; doi:10.7717/peerj.17641)

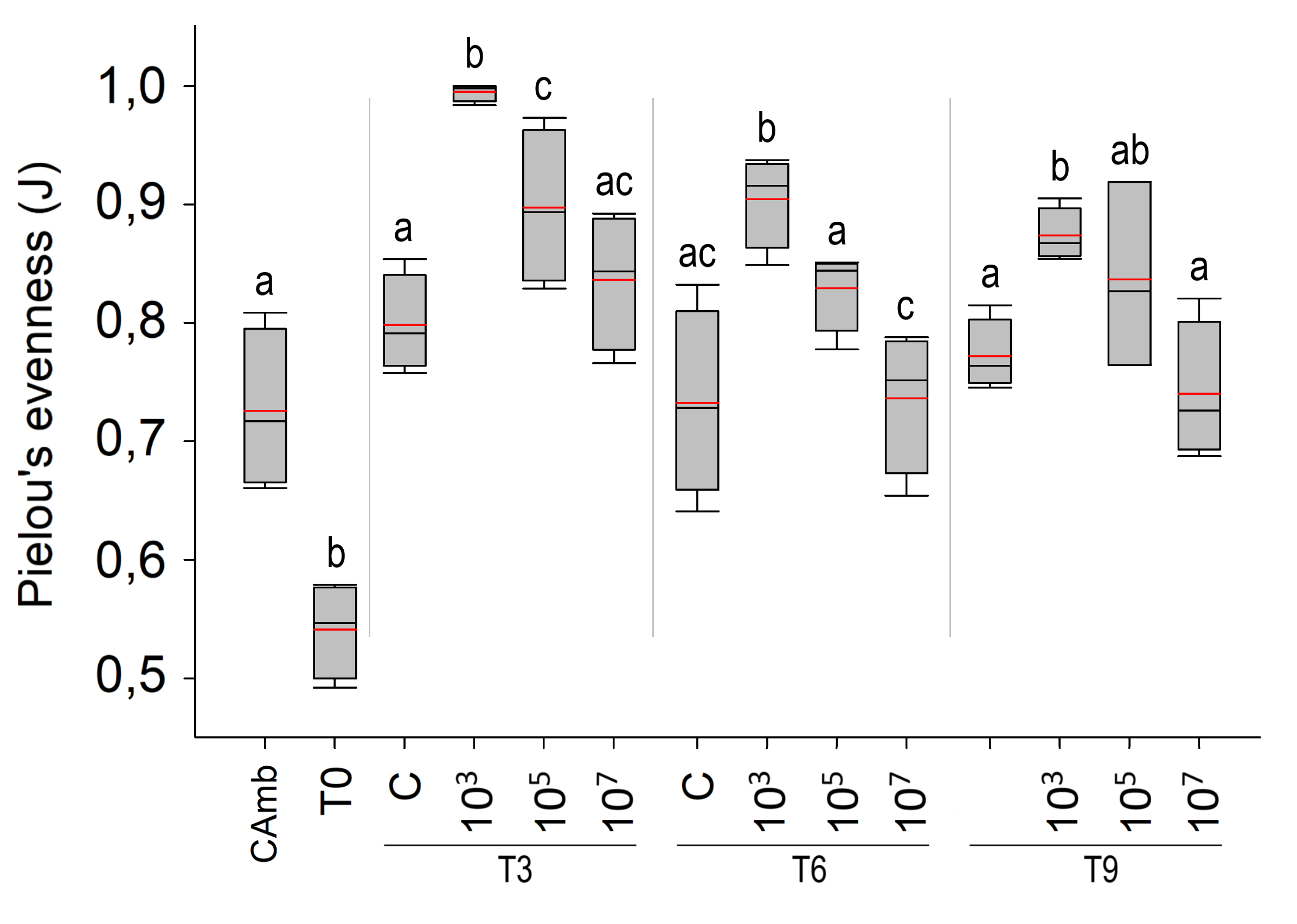

Supplement: Supplemental Information 1 — Mean Pielou index (red line) and median (black line) of meiofauna over time. Environmental control (Camb), Collection after 3 (T3), 6 (T6) and 9 (T9) days of exposure to polystyrene (PS). Different letters symbolize significant differences within each experimental time. Concentrations in particles/mL [file peerj-12-17641-s001.png]

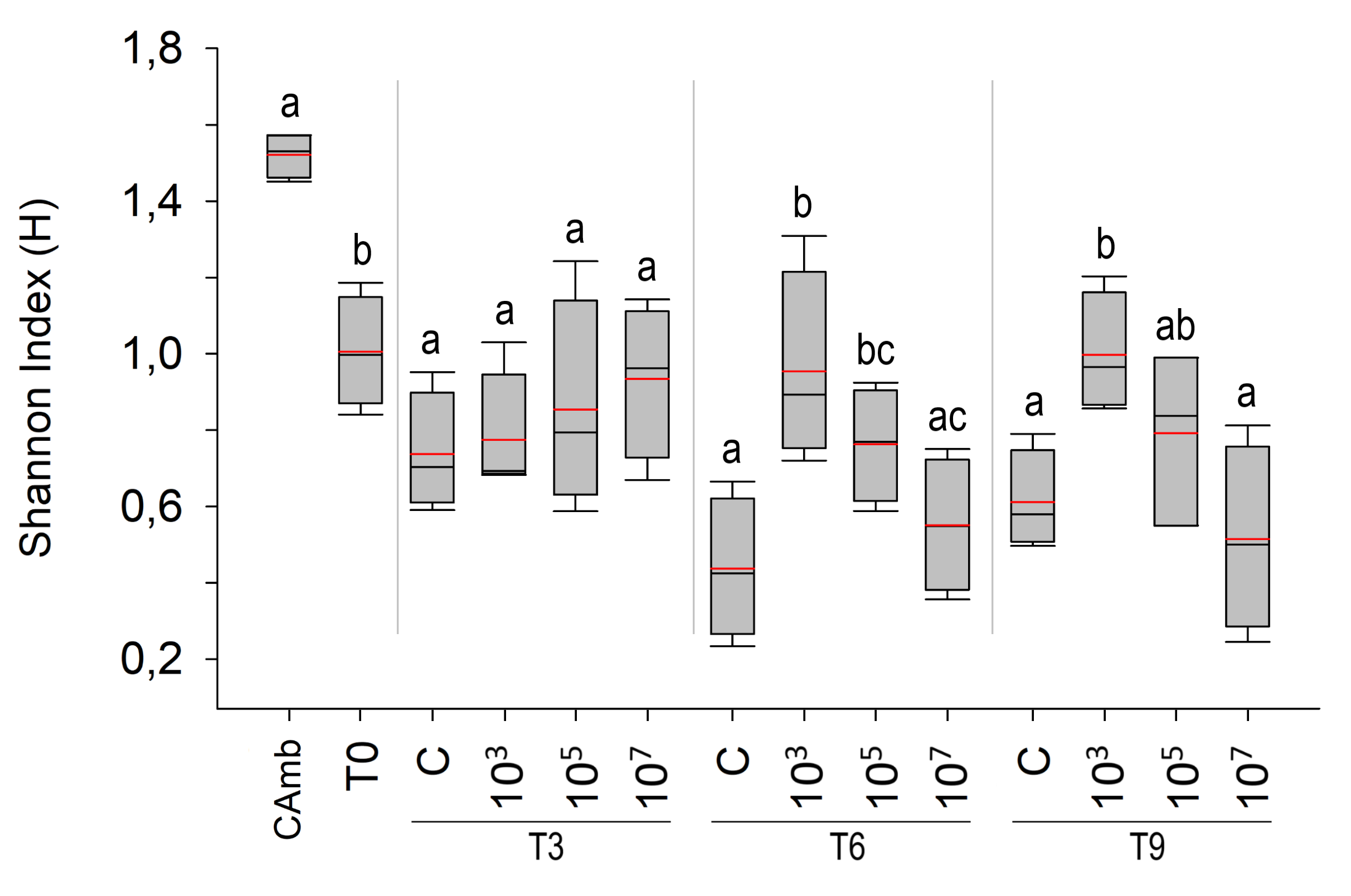

Supplement: Supplemental Information 2 — Mean Shannon index (red line) and median (black line) of meiofauna over time. Environmental control (Camb), Collection after 3 (T3), 6 (T6) and 9 (T9) days of exposure to polystyrene (PS). Different letters symbolize significant differences within each experimental time. Concentrations in particles/mL. [file peerj-12-17641-s002.png]

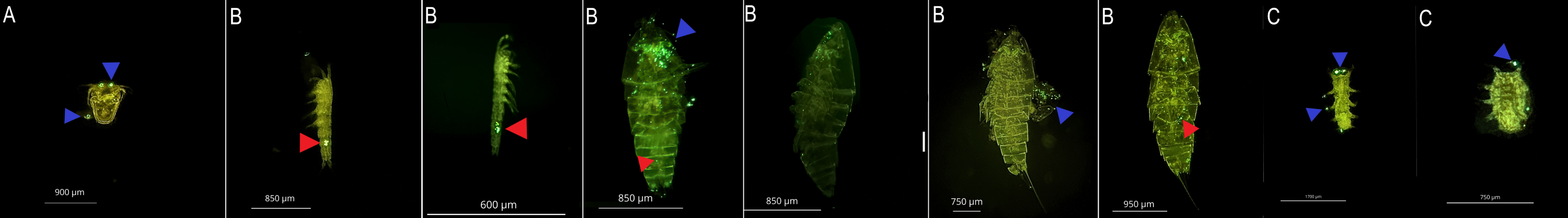

Supplement: Supplemental Information 3 — Internal (Ingestion-Red triangle) and external (Blue-triangle) contamination by 1 µm PS microplastic at a concentracion 107 part./mL. in marine meiofauna. [file peerj-12-17641-s003.png]

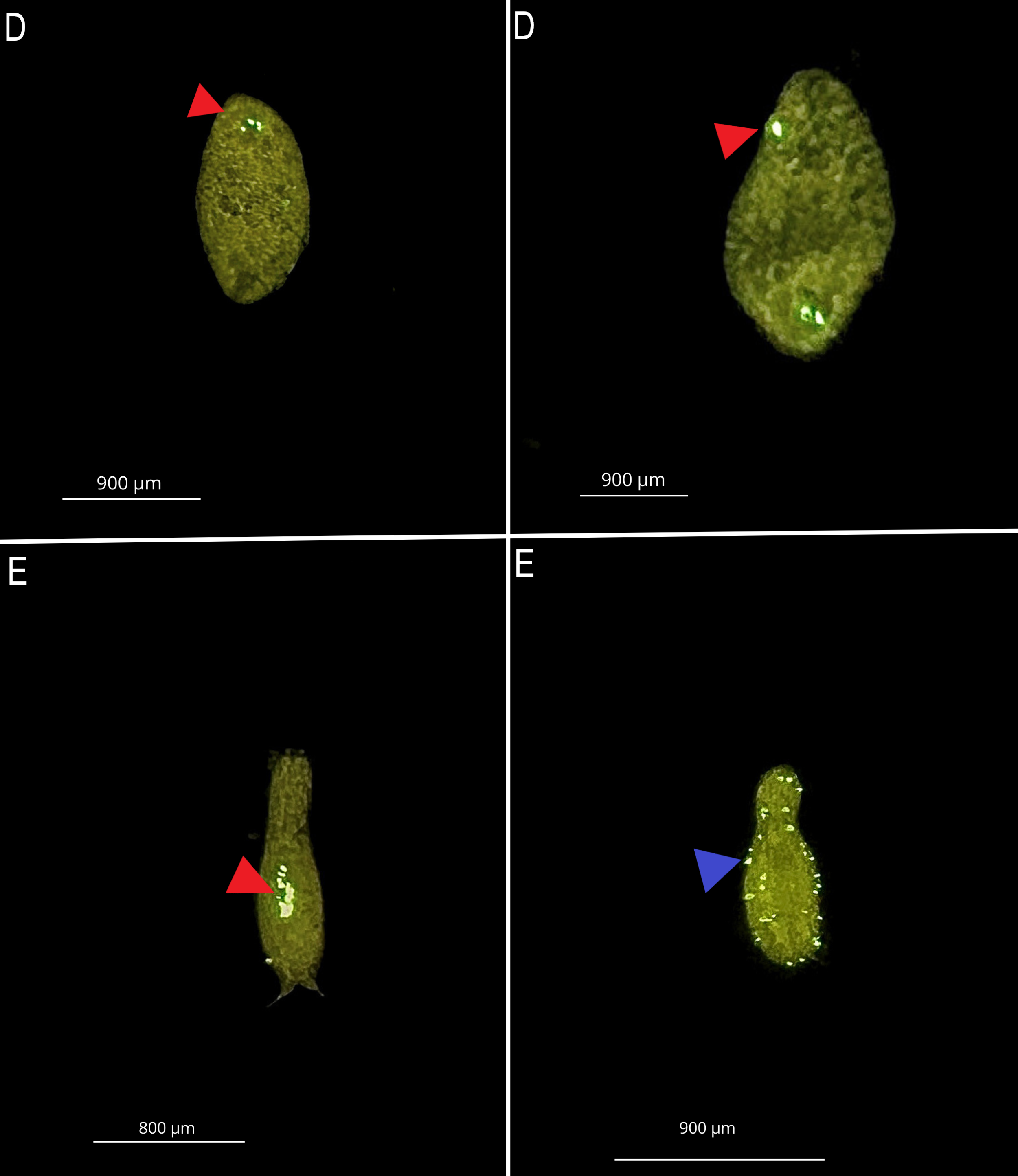

Supplement: Supplemental Information 4 — Internal (Ingestion-Red triangle) and external (Blue-triangle) contamination by 1 µm PS microplastic at a concentracion 107 part./mL. in marine meiofauna. [file peerj-12-17641-s004.png]

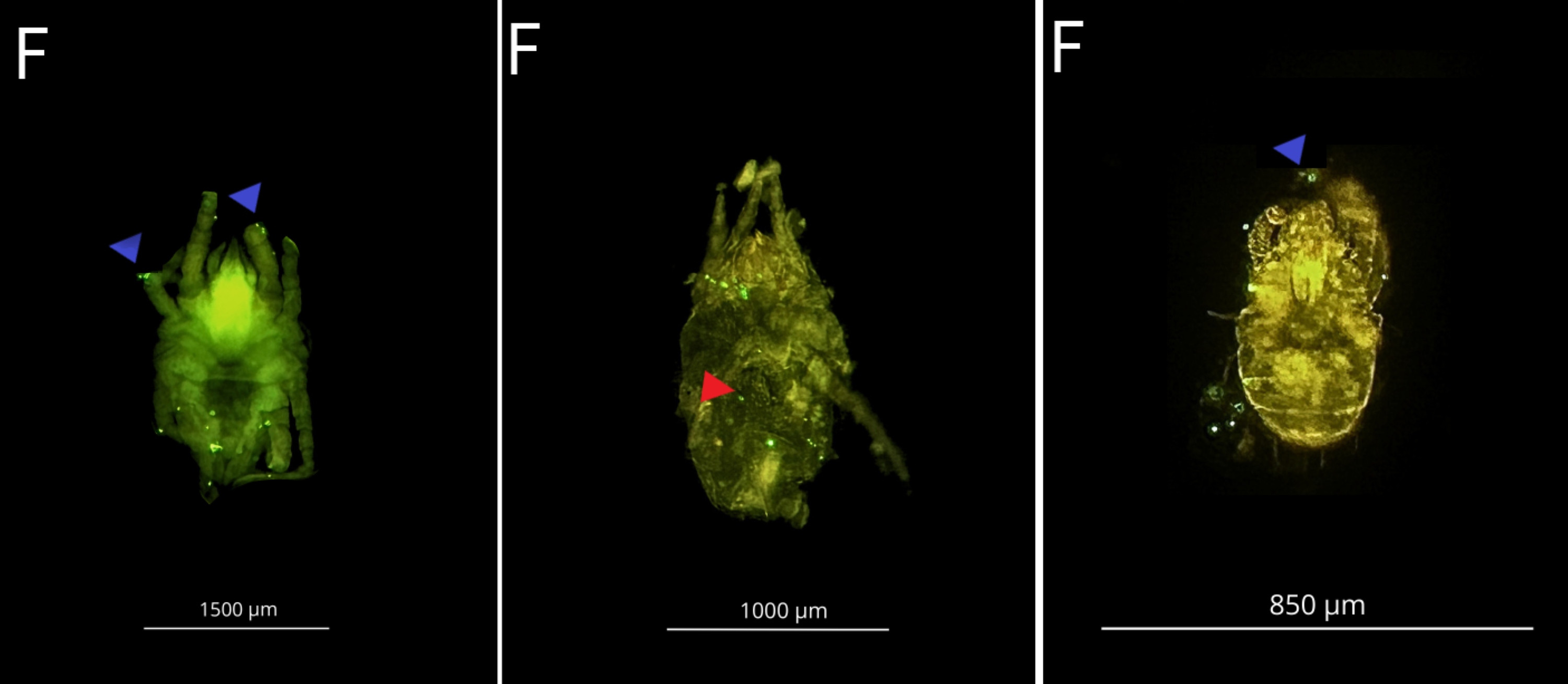

Supplement: Supplemental Information 5 — Internal (Ingestion-Red triangle) and external (Blue-triangle) contamination by 1 µm PS microplastic at a concentracion 107 part./mL. in marine meiofauna. [file peerj-12-17641-s005.png]

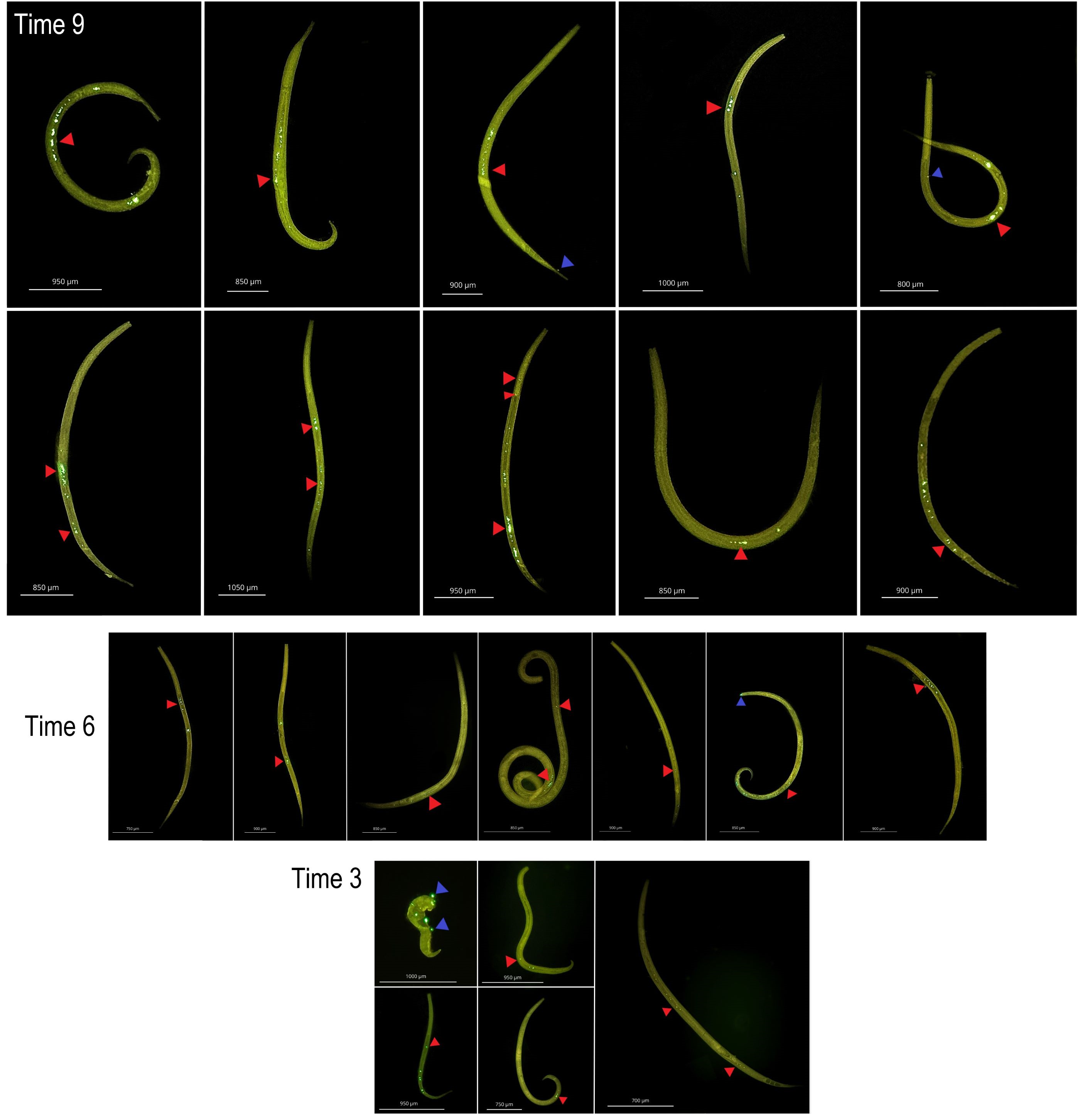

Supplement: Supplemental Information 6 — Internal (Ingestion-Red triangle) and external (Blue triangle) contamination in Nematoda over time at a concentracion 107part./mL. Three days after exposure to microplastic (Time 3), six days after exposure to microplastic (Time 6) and nine days after exposure to microplastic (Time 9). [file peerj-12-17641-s006.png]

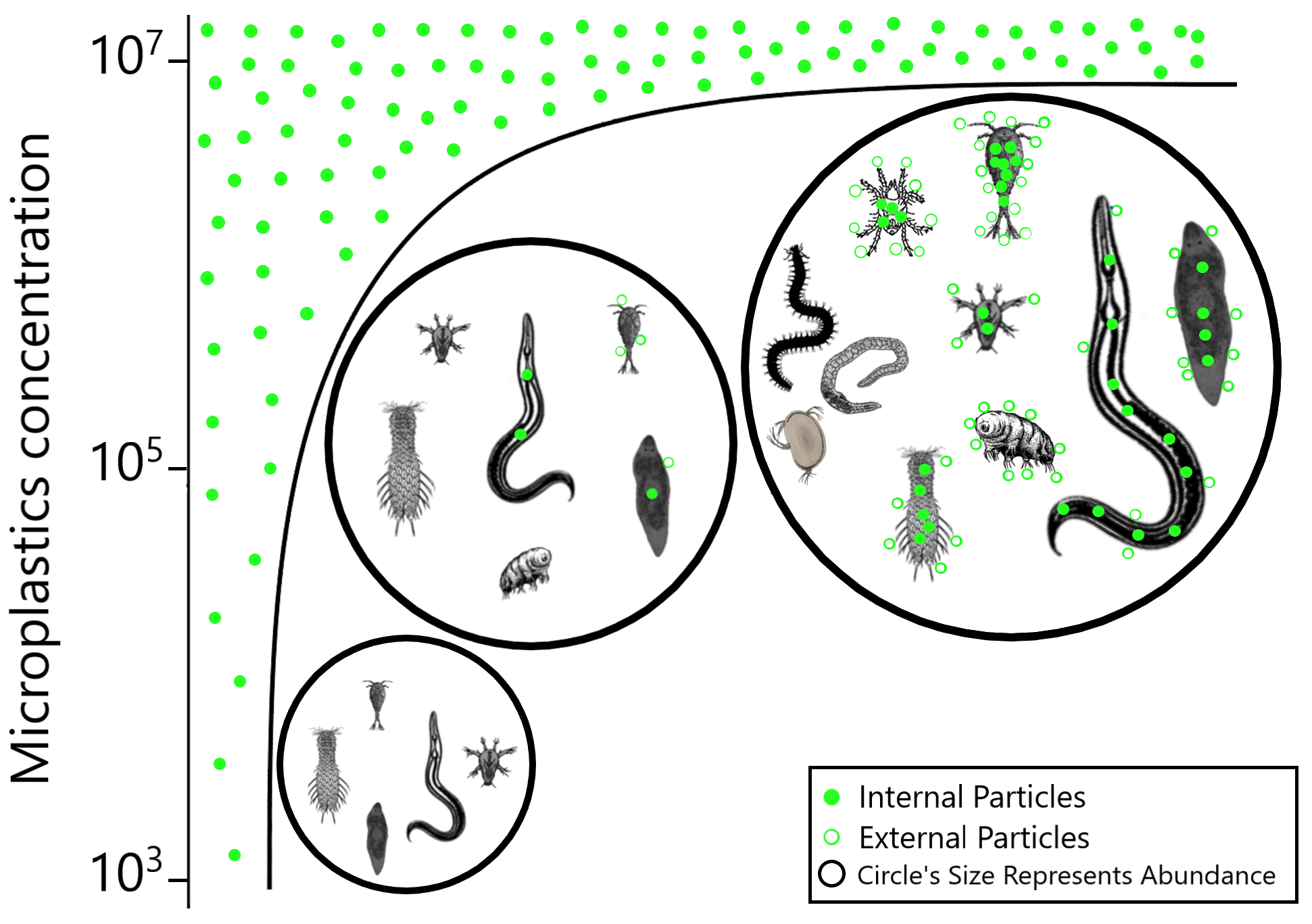

Supplement: Supplemental Information 8 [file peerj-12-17641-s008.png]
